# High endemicity in aquatic dance flies of Corsica, France (Diptera, Empididae, Clinocerinae and Hemerodromiinae), with the description of a new species of *Chelipoda*

**DOI:** 10.3897/zookeys.1039.66493

**Published:** 2021-05-25

**Authors:** Marija Ivković, Marija Perović, Patrick Grootaert, Marc Pollet

**Affiliations:** 1 Division of Zoology, Department of Biology, Faculty of Science, University of Zagreb, Rooseveltov trg 6, 10000, Zagreb, Croatia University of Zagreb Zagreb Croatia; 2 Entomology Unit, Royal Belgian Institute of Natural Sciences, Rue Vautier 29, B-1000, Brussels, Belgium Royal Belgian Institute for Natural Sciences (RBINS) Brussels Belgium; 3 Research Institute for Nature and Forest (INBO), Herman Teirlinckgebouw, Havenlaan 88 bus 73, B-1000, Brussels, Belgium Research Institute for Nature and Forest (INBO) Brussels Belgium

**Keywords:** Distribution, Empididae, endemicity, new species, Our Planet Reviewed expedition

## Abstract

All known records of aquatic dance flies (Empididae, Clinocerinae: 21 species; Hemerodromiinae: eight species) from the island of Corsica (France) are summarized, including previously unpublished data and data on the newly described species *Chelipoda puschae* Ivković, Perović & Grootaert, **sp. nov.** This species was collected during the “La Planète Revisitée Corsica 2019” survey and represents the first description of a new species in the genus *Chelipoda* from the European–Mediterranean region in more than 180 years. A key to European species of *Chelipoda* is provided. Including the new species, five species are recorded from Corsica for the first time: *Dolichocephala malickyi* Wagner, 1995, *Dolichocephala oblongoguttata* (Dale, 1878), *Dolichocephala ocellata* (Costa, 1854), *Chelifera subangusta* Collin, 1961, and *Hemerodromia unilineata* Zetterstedt, 1842. The new species is described and illustrated, and new records of aquatic dance flies from Corsica are given, with new data on 17 species in eight different genera. At present, 29 species of aquatic dance flies are known from Corsica, with 10 species endemic to the island.

## Introduction

The island of Corsica is situated in the Tyrrhenian Sea, about 170 km south of mainland France, about 90 km west of Italy, and separated from Sardinia by the Strait of Bonifacio. Mountains cover about two-thirds of the island, forming a single chain that runs in a north–south direction. Corsica is one of the most important centres of endemism for freshwater invertebrates in Europe (Giudicelli 1975; Ketmaier and Caccone 2013). In terms of its area of about 8700 km^2^, the concentration of endemic species on Corsica is one of the highest in Europe, with most of the endemic species located in spring brooks and streams at higher altitudes (Giudicelli 1975).

The aquatic Empididae (Hemerodromiinae and Clinocerinae) of Corsica have previously been studied on a number of occasions (Becker et al. 1910; Vaillant 1965, 1982; Wagner 1995). Becker et al. (1910) and Vaillant (1965, 1982) described, in total, three new species of aquatic dance flies from the island. Pusch (1996) provided the most detailed study of the Clinocerinae of Corsica, describing six new species. At present 23 species of aquatic dance flies are known from Corsica (Becker et al. 1910; Vaillant 1964, 1981; Wagner 1995; Pusch 1996), with nine endemics (Yang et al. 2007).

Both larval and adult aquatic Empididae are predators, mainly feeding on smaller aquatic dipterans such as Chironomidae, Simuliidae, and Psychodidae (Vaillant 1952, 1967; Harkrider 2000; Werner and Pont 2003; Ivković et al. 2007; Ivković and Plant 2015). Adult Hemerodromiinae are easily distinguished from adult Clinocerinae by their raptorial forelegs. They live and hunt in riparian vegetation, whereas adult Clinocerinae are primarily found on the surface of emergent wet stones or in moss mats (Ivković et al. 2007; Sinclair 2008).

Distribution and biodiversity studies are crucial for an understanding of the drivers of biodiversity hotspots (Ivković and Plant 2015; Schmidt-Kloiber et al. 2017). Regional distribution and biodiversity surveys are important for defining the biogeographic distribution of certain species or genera. They also contribute to the study of the various factors that influence changes in biodiversity and that subsequently affect the species conservation status (Meyer and Wagner 2011; Ivković et al. 2013a, 2017, 2020; Shamshev and Ivković 2020).

In this paper, we present new records of aquatic dance flies from Corsica (France) and also describe a new species. Detailed distribution data are presented, all resulting from the examination of specimens collected at 26 sites, sampled during the “La Planète Revisitée Corsica 2019” survey in June 2019.

## Materials and methods

### New specimen records

This paper is largely based on data and specimens obtained during the “La Planète Revisitée Corsica 2019” survey (http://laplaneterevisitee-corse.mnhn.fr/fr/participants-volet-terrestre-2019). This 6^th^ section of the large-scale biodiversity programme “La Planète Revisitée” or “Our Planet Reviewed” was organized solely by the French National Museum of Natural History (MNHN, Paris). Its primary aim is to rehabilitate taxonomic work that focuses on the largely neglected components of global biodiversity, i.e. invertebrates (both marine and terrestrial). The Corsica survey started in the spring of 2019 and will be concluded during 2021. It has entailed a number of blitz visits of one or two weeks to particular areas, and traps that were operational throughout the season. At the end of June 2019, a team of 10 French and two Belgian researchers conducted fieldwork in the Alta Rocca region in the south, and the Tartagine valley in the north. They employed a large number of sampling techniques including Malaise traps, pan traps of different colours, polytraps, light traps, pitfall traps, and Lindgren funnel traps. Sweep nets and hand collecting were also used. Between June 23 and 26, 2019, 17 sites at four different research locations in the Alta Rocca area (southern Corsica) were selected for pan trap sampling by Marc Pollet. At three locations, four sampling sites were operational and at the main research location, Campu di Bonza (BO), a fifth sampling site was added. In nearly all sampling sites the same sampling strategy was applied: five blue, five yellow and five white pan traps were installed at soil surface level, in five 3-coloured trap sets. They were filled to two-thirds full with a light formalin solution (<5%) and detergent to lower the surface tension. All traps were operational for four consecutive days (27–30 June 2019). A total of 258 pan traps were in operation during this period. In addition, at each of the sampling sites (and also at other places in each location), flies were collected by sweep net and by hand (with a small polymer jar). All specimens included in the present paper were retrieved from the pan trap and sweep net samples, and from the hand collecting.

All sampling sites were georeferenced while sampling. The names of taxa reflect current nomenclature and classifications (Sinclair 1995; Yang et al. 2007). Species of *Wiedemannia* mentioned herein are not assigned to subgenus, as the subgenera do not represent monophyletic groups and are therefore considered invalid (Ivković et al. 2019). The literature used for identification included Engel (1939, 1940), Vaillant (1965, 1982), Wagner and Horvat (1993), Wagner (1995), and Pusch (1996).

**Figure 1.**
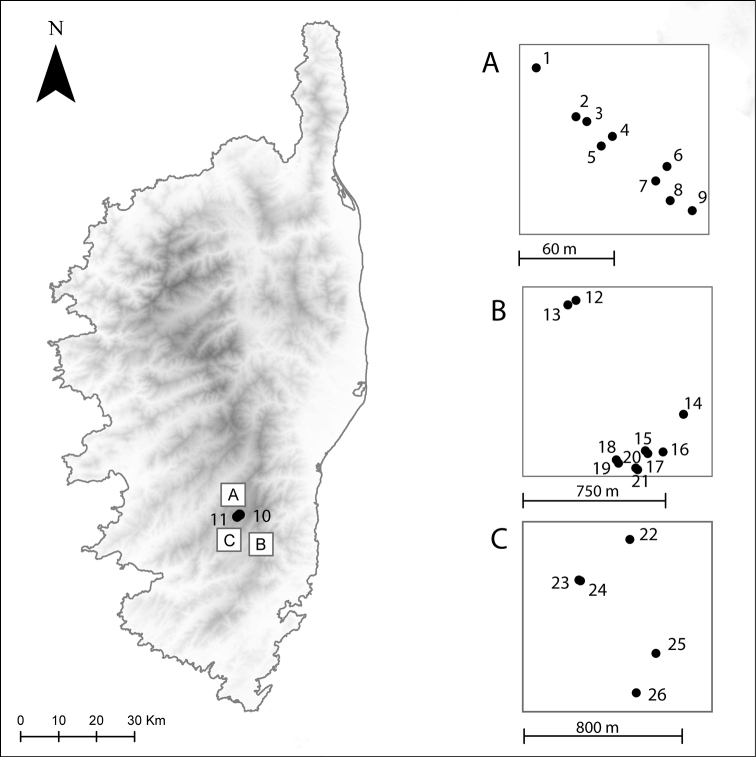
Sampling sites on Corsica (France) as part of the “La Planète Revisitée Corsica 2019” expedition, where aquatic Empididae were encountered during June 2019 (See Table 1 for codes) **A** Zicavo, Ponte di Valpine **B** Zonza, Samulaghia **C** Serra di Scopamène et Sorbollano, Campu di Bonza; 10, 11: Serra di Scopamène, Castellu d’Ornucciu; detailed position of sampling sites 10 and 11 not given in separate box.

Records are listed for each species. A list of sampling sites with latitude, longitude, altitude, and collecting method is presented in Table 1, and a map showing the positions of the georeferenced sampling sites is also provided (Fig. 1). The collected aquatic dance flies were preserved in 75% ethanol solution (EtOH). For identification purposes, in some cases male terminalia were macerated in hot 85% lactic acid, dissected, and stored in 75% ethanol along with the specimen in the same tube. All specimens listed in the Material examined sections were collected by Anja De Braekeleer, Claire Villemant, and Marc Pollet. Taxonomic diversity is considered at the level of subfamily, genus, and species. Label data for primary types are cited in full, with original spelling, punctuation, and dates. This study is based on material housed in the following institutions: National Museum of Natural History, Paris, France (**MNHN**); Royal Belgian Institute of Natural Sciences, Brussels, Belgium (**RBINS**); col. M. Ivković, University of Zagreb, Croatia (**UZC**); and Canadian National Collection of Insects, Ottawa, Canada (**CNC**). Terminology for adult structures primarily follows Cumming and Wood (2017). The femoral formula is taken from Plant (2009). Homologies of the male terminalia follow those of Sinclair and Cumming (2006) and Plant (2009).

**Table 1.** List of sampling sites in Corsica during the “La Planète Revisitée Corsica 2019” survey. MSW = random sweep netting, SW = visual sweep netting, HC = collecting by hand, BPT = blue pan traps, YPT = yellow pan traps, WPT = white pan traps.

ID	Location	Coolecting date	Latitude / Longitude	Altitude (m)	Type of method
**Zicavo, Ponte di Valpine**					
**1**	Zicavo, Ponte di Valpine, at waterfall in riverbed	25.vi.2019	41°52'29.0"N, 09°08'04.7"E	1264	SW
**2**	Zicavo, Ponte di Valpine, near small waterfall in riverbed	29.vi.2019	41°52'28.0"N, 09°08'05.8"E	1271	HC
**3**	Zicavo, Ponte di Valpine, in splash zone of rocks in riverbed	25.vi.2019	41°52'27.9"N, 09°08'06.1"E	1270	HC
**4**	Zicavo, Ponte di Valpine, on dry rocks and on seeps on rocks in riverbed	29.vi.2019	41°52'27.6"N, 09°08'06.8"E	1277	HC
**5**	Zicavo, Ponte di Valpine, on rocks in riverbed	25–29.vi.2019	41°52'27.4"N, 09°08'06.5"E	1282	BPT, YPT
**6**	Zicavo, Ponte di Valpine, on rocks in riverbed	25–29.vi.2019	41°52'27.0"N, 09°08'08.3"E	1283	BPT, YPT
**7**	Zicavo, Ponte di Valpine, on rocks in riverbed	25.vi.2019	41°52'26.7"N, 09°08'08.0"E	1287	SW
**8**	Zicavo, Ponte di Valpine, at seep on beech forest slope	25–29.vi.2019	41°52'26.3"N, 09°08'08.4"E	1286	YPT
**9**	Zicavo, Ponte di Valpine, on rocks on beech forest slope	25–29.vi.2019	41°52'26.1"N, 09°08'09.0"E	1298	WPT
**Serra di Scopamène, Castellu d’Ornucciu**					
**10**	Serra di Scopamène, Castellu d’Ornucciu, in higher Alnus forest	26–30.vi.2019	41°49'58.6"N, 09°09'26.1"E	1580	YPT
**11**	Serra di Scopamène, Castellu d’Ornucciu, in shady sites along stream in pozzine landscape	26–30.vi.2019	41°50'00.5"N, 09°09'27.6"E	1568	YPT, WPT
**Zonza, Samulaghia**					
**12**	Zonza, Samulaghia, on rocks at small waterfall on stream	24.vi.2019	41°46'08.07"N, 09°13'22.86"E	1116	SW
**13**	Zonza, Samulaghia, canopied seep along the road at edge of forest	24.vi.2019	41°46'07.23"N, 09°13'20.92"E	1093	MSW
**14**	Zonza, Samulaghia, sapinière forest (soil surface)	24–28.vi.2019	41°45'48.61"N, 09°13'47.56"E	1363	YPT
**15**	Zonza, Samulaghia, on dry rocks near seep in sapinière forest	24–28.vi.2019	41°45'42.30"N, 09°13'39.01"E	1208	BPT, YPT, WPT
**16**	Zonza, Samulaghia, sapinière forest	24–28.vi.2019	41°45'42.13"N, 09°13'43.06"E	1267	YPT
**17**	Zonza, Samulaghia, in dry sapinière forest	24–28.vi.2019	41°45'41.78"N, 09°13'39.52"E	1209	YPT
**18**	Zonza, Samulaghia, on rocky seep in Sapinière forest (edge of forest)	24–28.vi.2019	41°45'40.1"N, 09°13'32.9"E	1231	YPT
**19**	Zonza, Samulaghia, seep on rocks in sapinière forest	28.vi.2019	41°45'40.1"N, 09°13'32.9"E	1188	HC
**20**	Zonza, Samulaghia, marshy seep in dry sapinière forest	24–28.vi.2019	41°45'39.6"N, 09°13'37.2"E	1244	BPT, YPT, WPT, MSW
**21**	Zonza, Samulaghia, on low vegetation in marshy seep in sapinière forest	24.vi.2019	41°45'39.3"N, 09°13'36.8"E	1243	MSW
**Serra di Scopamène et Sorbollano, Campu di Bonza**					
**22**	Serra di Scopamène et Sorbollano, Campu di Bonza, on banks of river in oak forest	23–27.vi.2019	41°46'28.3"N, 09°07'26.9"E	845	BPT, YPT, WPT
**23**	Serra di Scopamène et Sorbollano, Campu di Bonza, on gravelly muddy seep in deciduous forest	23–27.vi.2019	41°46'21.5"N, 09°07'15.8"E	920	BPT
**24**	Serra di Scopamène et Sorbollano, Campu di Bonza, on gravelly muddy seep in deciduous forest	23–27.vi.2019	41°46'21.4"N, 09°07'16.2"E	935	YPT
**25**	Serra di Scopamène et Sorbollano, Campu di Bonza, edge of oak forest	27.vi.2019	41°46'09.55"N, 09°07'32.83"E	919	YPT
**26**	Serra di Scopamène et Sorbollano, Campu di Bonza, clearing in oak forest	23.vi.2019	41°46'03.08"N, 09°07'28.58"E	911	SW

### Data analysis

A list of species was compiled from all specimen data collected during this survey and from all available literature data (Table 2). The distribution range of the species was constructed by assembling information from species lists by Becker et al. (1910), Vaillant (1965, 1982) Wagner (1995), Pusch (1996), Chvála (2012), and Yang et al. (2007). The zoogeographic categorization of species was conducted according to Vigna Taglianti et al. (1999).

**Table 2.** List of aquatic dance flies (Diptera: Empididae, Clinocerinae, Hemerodromiinae) of Corsica, with a summary of their distribution range. Species recorded here for the first time from Corsica are listed with “*”.

Species	Distribution range
** Clinocerinae **	
*Clinocera appendiculata* (Zetterstedt, 1838)	European
*Clinocera nigra* Meigen, 1804	West Palaearctic
*Clinocera stagnalis* (Haliday, 1833)	Holarctic
*Clinocerella gereckei* (Wagner & Horvat, 1993)	Corsica (France), Sardinia (Italy)
*Clinocerella wagneri* (Pusch, 1996)	Corsica (France)
*Dolichocephala guttata* (Haliday, 1833)	European
*Dolichocephala malickyi Wagner, 1995	Mediterranean (Tunisia, Spain, Corsica (France))
*Dolichocephala oblongoguttata (Dále, 1878)	European
*Dolichocephala ocellata (Costa, 1854)	European-Mediterranean
*Kowarzia barbatula* (Mik, 1880)	South European
*Kowarzia bipunctata* (Haliday, 1833)	European-Mediterranean
*Kowarzia cataractae* (Pusch, 1996)	Corsica (France)
*Kowarzia schnabli* Becker, 1910	Corsica (France)
*Kowarzia tibiella* (Mik, 1880)	Central European
*Wiedemannia ariolae* Pusch, 1996	Corsica (France)
*Wiedemannia bravonae* Pusch, 1996	Corsica (France)
*Wiedemannia corsicana* Vaillant, 1964	Corsica (France)
*Wiedemannia czernyi* (Bezzi, 1905)	Mediterranean (Corsica (France), Greece, Italy)
*Wiedemannia kallistes* Pusch, 1996	Corsica (France)
*Wiedemannia martini* Pusch, 1996	Corsica (France)
*Wiedemannia rhynchops* (Nowicki, 1868)	Central European
** Hemerodromiinae **	
*Chelifera barbarica* Vaillant, 1981	Mediterranean (Algeria, France (Corsica), Greece (Dodecanese Is.))
*Chelifera corsicana* Vaillant, 1981	Corsica (France)
*Chelifera precatoria* (Fallén, 1816)	European
*Chelifera subangusta Collin, 1961	European
*Chelipoda albiseta* (Zetterstedt, 1838)	European
*Chelipoda vocatoria* (Fallen, 1816)	European
*Chelipoda puschae Ivković, Perović & Grootaert, sp. nov.	Corsica (France)
*Hemerodromia unilineata Zetterstedt, 1842	European

## Results

### Taxonomy

#### Chelipoda puschae

Taxon classificationAnimaliaDipteraEmpididae

Ivković, Perović & Grootaert sp. nov.

7F3494AA-1E17-5BA5-8ADE-9239C5FC32D8

http://zoobank.org/2F661C1D-B83B-47D7-831B-B1B0444579F7

Figures 2 , 3 , 4

##### Type locality.

France, Corsica, Zonza, Samulaghia, in dry sapinière forest, 41°45'41.78"N, 09°13'39.52"E

##### Type material.

***Holotype*** • 1 ♂, labelled: “FRANCE, CORSICA; FR-COR/2019/096 (sample code); La Planète Revisitée – MNHN Corsica / 2019; Zonza, Samulaghia; in dry sapinière forest; 41°45'41.78"N, 09°13'39.52"E; 24–28.vi.2019; M. Pollet leg.”; HOLOTYPE/*Chelipoda pusche* Ivković, Perović & Grootaert” (MNHN, in 80% ethanol). ***Paratypes*** same data as holotype (• 10 ♂♂, 10 ♀♀, MNHN; • 63 ♂♂, 29 ♀♀, UZC; • 45 ♂♂, 14 ♀♀; RBINS; • 55 ♂♂, 24 ♀♀, CNC).

##### Additional material.

See section with all other records of aquatic empidids.

##### Diagnosis.

Small, slender brown species with black head, darker median stripe on thorax and yellow legs; upper lobe of cercus slightly curved and pointed; subepandrial process sharply projecting anteriorly, rather slim and straight.

##### Description.

**Figure 2.**
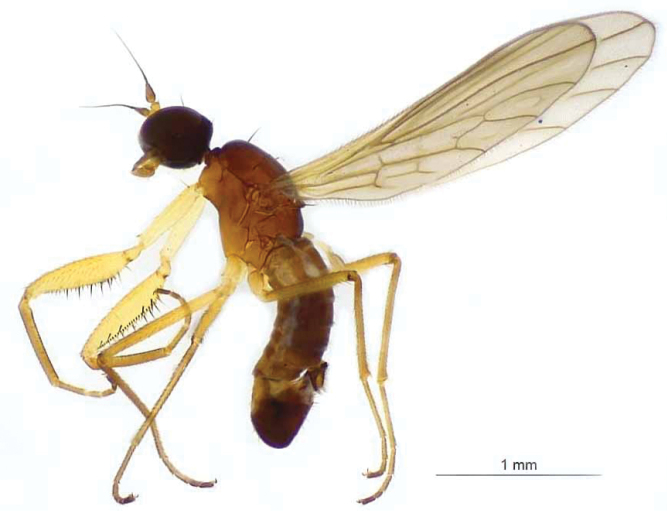
Male (not holotype) of *Chelipoda puschae* Ivković, Perović & Grootaert, sp. nov.

**Figure 3.**
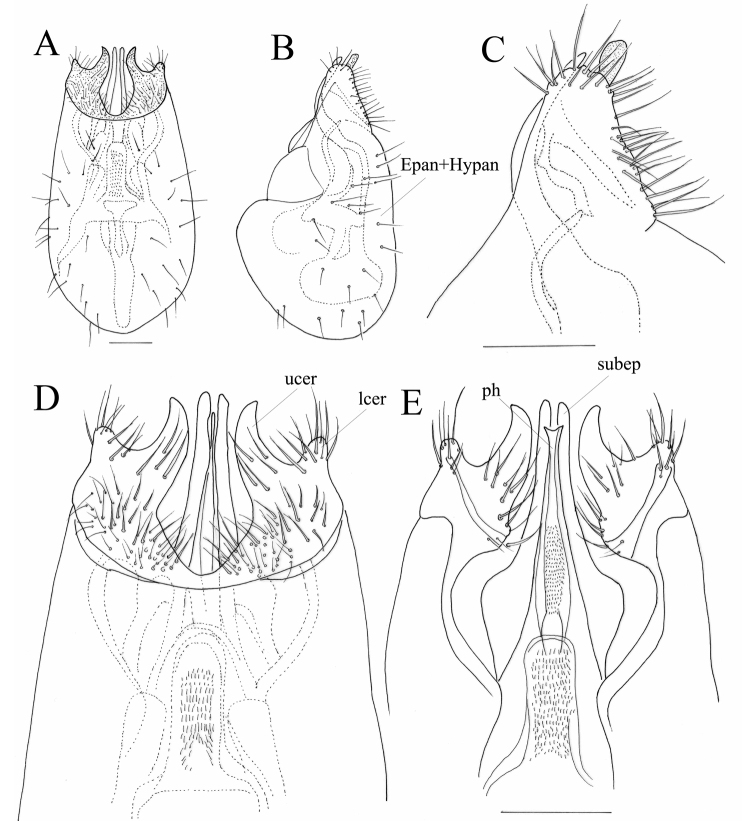
Male terminalia of *Chelipoda puschae* Ivković, Perović & Grootaert, sp. nov. **A** ventral view **B** lateral view **C** lateral view **D** ventral view **E** dorsal view **C–E** show details of the apex. Abbreviations: Epan+Hypan, fused epandrium and hypandrium; lcer, lower lobe of cercus; ucer, upper lobe of cercus; subep, subepandrial process; ph, phallus. Scale bars: 0.1 mm.

**Male** (Figs 2, 3) Body length (based upon 10 specimens): 2.6–2.9 mm; wing length: 2.6–2.9 mm. Head black, with strong black setae, including 2 ocellar setae, outer vertical setae and 4 postocular setae, other setae fine and paler; patch of fine setae posterior of mouth. Mouthparts dark yellow. Eyes black, almost touching below antenna. Antennae, scape, and pedicel yellow, pedicel twice as long as scape; postpedicel light brown, twice as long as pedicel. Arista-like stylus light brown, about 4× as long as postpedicel.

Sternum yellow, with dark yellow pleura and light brown scutum. Dark brown longitudinal stripe in centre of scutum dorsally broadening towards pronotum and scutellum. Setae on scutum black, with 2 pairs of acrostichal setae, middle pair stronger, posterior pair rather fine and close to scutellum. One anterior pair and one posterior pair of dorsocentral setae, both long and strong. Three notopleural setae, upper posterior rather strong, others smaller and thinner. One pair of strong, marginal scutellar setae.

Legs light yellow, with tarsomeres 4 and 5 darker. Fore coxa with 2 basal setae, upper longer and stronger than lower. Fore tibia slightly longer than fore coxa, distinctly inflated. Femoral formula of fore leg (based upon 10 specimens): 6 anteroventral spines (range 5 or 6), 27 anteroventral denticles (range 23–28), 13 posteroventral denticles (range 10–14), 7 posteroventral spines (range 5–8) and 1 basal spine. All spines dark brown, denticles black. Tibia of a foreleg almost as long as femur.

Wing membrane transparent, veins light brown. Squamae with black fringe. Halter pale brown.

Abdominal tergites and sternites brown, tergites darker than sternites, with short setae, dark on tergites, paler on sternites.

Male terminalia (Fig. 3): blackish, darker on upper lobe of cercus, visible part of phallus yellowish. Epandrium and hypandrium fused, rather rounded in lateral view, bearing scattered small dark setae. Left and right lamellae separated by unpigmented densely micropilose membrane. Cercus fused with epandrium + hypandrium, forked, upper lobe of cercus slightly curved and pointed. Subepandrial process sharply projecting anteriorly, rather slim and straight. Phallus apically slender, yellowish.

**Figure 4.**
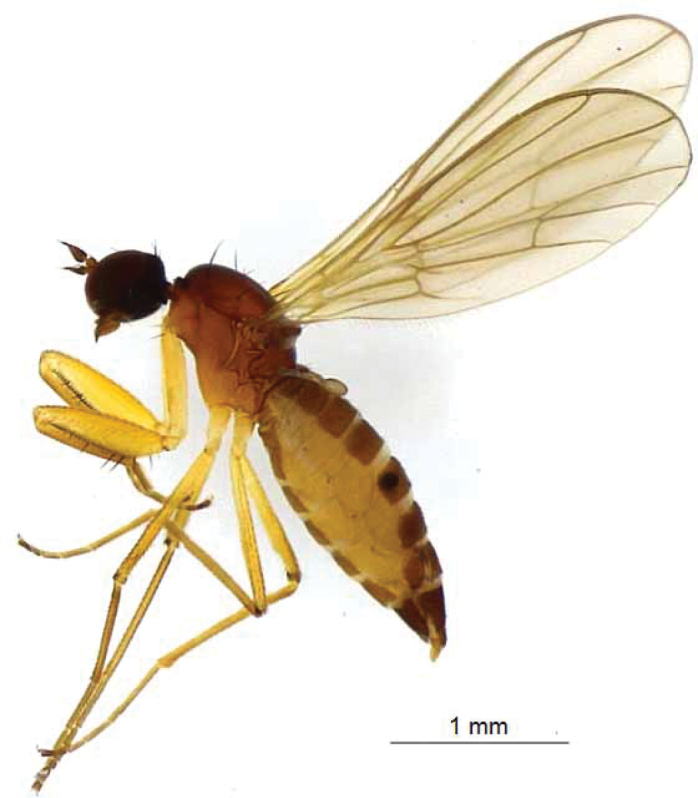
Female of *Chelipoda puschae* Ivković, Perović & Grootaert, sp. nov.

**Female.** (Fig. 4) Similar to male, except: antenna darker; femoral spines longer and stronger.

##### Etymology.

The species is named after the German entomologist Martina Pusch, who described six species of Empididae (Clinocerinae) from Corsica.

##### Remarks.

**Figure 5.**
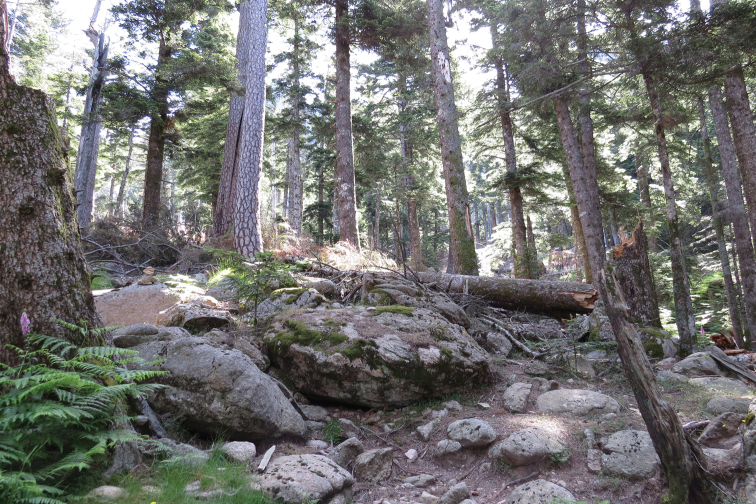
The pine forest (sapinière) at Zonza, Samulaghia, in southern Corsica, investigated 24–28 June 2019 as part of the “La Planète Revisitée Corsica 2019” survey.

At present, this species is only known from Corsica. It was collected at each of the four localities and eight of the 17 sampling sites investigated during the “La Planète Revisitée Corsica 2019” survey, ranging from open pozzine landscapes to riverbanks in dry oak forests between 845 m and 1,580 m. *Chelipoda puschae* sp. nov. clearly prefers pine forest (sapinière) (Fig. 5) over the other biotopes sampled, with over 96% of the 387 specimens collected here. Within this forest, the species was collected in greatest numbers at a dry rocky site, where its abundance was over five times as high as in the other more humid sampling sites in the same location. Over 97% of all specimens in the pine forest were retrieved from yellow pan traps, and less than 3% from white and blue pan traps.

### Key to males of European species of *Chelipoda*

**Table:** 

1	Epandrium + hypandrium with dorsal claw like appendage pointing downwards; cercus in lateral view small and triangular	***Chelipoda vocatoria* (Fallén)**
–	Epandrium and hypandrium rather rounded, without appendages; cerci forked (Fig. 3A–E)	***Chelipoda puschae* sp. nov.**
–	Cerci rectangular or rhomboidal, with or without elongate dorsal appendage	**2**
2	Cerci elongate in lateral view, with strong dorsal appendage bearing 3 distal teeth-like projections	***Chelipoda inexpectata* Tuomikoski**
–	Cerci rhomboidal in lateral view, with inner lobes pointing towards one another in dorsal view	***Chelipoda albiseta* (Zetterstedt)**

## New records of aquatic Empididae (Clinocerinae & Hemerodromiinae) from Corsica (France)

The following format is used for the distribution data: Material examined: number of males (♂) and/or females (♀), locality and location name, description of sampling site, collection date or period, collecting method (sampling site ID, see Table 1). Species recorded for the first time for Corsica (France) are indicated with “*” in front of the species names. A full list of sampling sites is given in Table 1.

### Subfamily Clinocerinae

#### Clinocera nigra

Taxon classificationAnimaliaDipteraEmpididae

Meigen, 1804

4BF07BF2-650F-569F-B8CE-33DC1A79E725

##### Material examined.

• 1♂; Zicavo, Ponte di Valpine, on dry rocks and on seepages on rocks in riverbed; 29.vi.2019; HC (4).

##### Remarks.

Previously reported by Becker et al. (1910) and Pusch (1996).

#### Clinocerella wagneri

Taxon classificationAnimaliaDipteraEmpididae

(Pusch, 1996)

95D1B228-338C-5E9A-A361-CD2AE1D556BB

##### Material examined.

• 1♂; Serra di Scopamène et Sorbollano, Campu di Bonza, on gravelly muddy seepage in deciduous forest; 23–27.vi.2019; BPT (23).

##### Remarks.

Recorded and described by Pusch (1996).

#### * Dolichocephala malickyi

Taxon classificationAnimaliaDipteraEmpididae

Wagner, 1995

AF23EB37-C9AD-5289-8208-7BC962E33A67

##### Material examined.

• 1♀; Serra di Scopamène, Castellu d’Ornucciu, in shady sites along stream in pozzine landscape, 26–30.vi.2019, WPT (11).

##### Remarks.

This is the first tentative record of this species for Corsica. Although the wing pattern corresponds exactly to that in Wagner (1995), as this is a female, the identification is not 100% certain. We thus await the discovery of the corresponding male.

#### * Dolichocephala oblongoguttata

Taxon classificationAnimaliaDipteraEmpididae

(Dale, 1878)

7CD22C6A-1424-57F5-979D-1B4B86AEF8F6

##### Material examined.

• 1♂; Zicavo, Ponte di Valpine, on rocks in riverbed; 25–29.vi.2019; YPT (5) • 1♂, 1♀; Zicavo, Ponte di Valpine, on rocks in riverbed; 25–29.vi.2019; YPT (6) • 1♂; Serra di Scopamène, Castellu d’Ornucciu, in shady sites along stream in pozzine landscape; 26–30.vi.2019; YPT (11) • 1♂; Zonza, Samulaghia, canopied seepage along road at edge of forest; 24.vi.2019; MSW (13).

##### Remarks.

This is the first record of this species for Corsica. Becker et al. (1910) reported *Dolichocephala guttata* (Haliday, 1833), but this record is doubtful as the wing patterns of both species are almost identical and females are indistinguishable. Unfortunately, there is no information on how many specimens of each sex were collected by Becker et al. (1910). As *D. guttata* and *D. oblongoguttata* can be easily confused and/or mixed, the occurrence of *D. guttata* in Corsica needs to be confirmed.

#### * Dolichocephala ocellata

Taxon classificationAnimaliaDipteraEmpididae

(Costa, 1854)

806D5501-BA31-56F3-9D27-80C2E1F22245

##### Material examined.

• 1♀; Serra di Scopamène, Castellu d’Ornucciu, in shady sites along stream in pozzine landscape; 26–30.vi.2019; YPT (11) • 4♂; Serra di Scopamène et Sorbollano, Campu di Bonza, on banks of river in oak forest; 23–27.vi.2019; BPT (22) • 1♂, 1♀; same data, WPT (22) • 1♂; same data, YPT (22).

##### Remarks.

This is the first record of this species for Corsica. Pusch (1996) reported a female of the *D. ocellata* group and we here confirm this record with male and female specimens.

#### Kowarzia bipunctata

Taxon classificationAnimaliaDipteraEmpididae

(Haliday, 1833)

F0EF44A1-BE35-5726-9048-32DF85458E26

##### Material examined.

• 1♂; Zonza, Samulaghia, on dry rocks near seepage in sapinière forest, 24–28.vi.2019, BPT (15).

##### Remarks.

Previously reported by Pusch (1996).

#### Kowarzia cataractae

Taxon classificationAnimaliaDipteraEmpididae

(Pusch, 1996)

83E7C7B8-C8B5-590F-BE3C-4289621DE301

##### Material examined.

• 2♂; Zonza, Samulaghia, on dry rocks near seepage in sapinière forest; 24–28.vi.2019; BPT (15).

##### Remarks.

Recorded and described by Pusch (1996).

#### Kowarzia schnabli

Taxon classificationAnimaliaDipteraEmpididae

Becker, 1910

1A048EC3-801C-5F94-8DAF-F00B7E041A78

##### Material examined.

• 1♂; Zicavo, Ponte di Valpine, on rocks in riverbed; 25–29.vi.2019; BPT (5) • 1♂; Zonza, Samulaghia, on rocks at small waterfall on stream; 24.vi.2019; SW (12) • 2♂; Zonza, Samulaghia, on dry rocks near seepage in sapinière forest; 24–28.vi.2019; BPT (15) • 1♂; Zonza, Samulaghia, marshy seepage in dry sapinière forest (20); 24–28.vi.2019; BPT (20).

##### Remarks.

This endemic species was described in Becker et al. (1910) and also collected by Pusch (1996).

#### Kowarzia tibiella

Taxon classificationAnimaliaDipteraEmpididae

(Mik, 1880)

0AF627E9-24B8-5187-AC9F-7C2A3BD4BBD5

##### Material examined.

• 1♂; Zonza, Samulaghia, on dry rocks near seepage in sapinière forest; 24–28.vi.2019; BPT (15) • 4♂, 4♀; Zonza, Samulaghia, seepage on rocks in sapinière forest; 28.vi.2019; HC (19).

##### Remarks.

Previously reported by Vaillant (1964) and again by Pusch (1996).

#### Wiedemannia corsicana

Taxon classificationAnimaliaDipteraEmpididae

Vaillant, 1964

91079C51-06FE-55D2-90F0-2A273336E996

##### Material examined.

• 1♂; Zicavo, Ponte di Valpine, on rocks in riverbed; 25.vi.2019; SW (7).

##### Remarks.

Described and recorded for the first time by Vaillant (1964), and also collected by Pusch (1996).

#### Wiedemannia czernyi

Taxon classificationAnimaliaDipteraEmpididae

(Bezzi, 1905)

A6F64E02-7201-5CFD-A519-C029E7A9FEE5

##### Material examined.

• 2♂, 6♀; Zicavo, Ponte di Valpine, at waterfall in riverbed; 25.vi.2019; SW (1) • 2♂, 9♀; Zicavo, Ponte di Valpine, on dry rocks and on seepages on rocks in riverbed; 29.vi.2019; HC (4).

##### Remarks.

This species was previously reported by Wagner (1995) and Pusch (1996). This species was reported in Becker et al. (1910) as *Röederia longipennis* Mik, 1880, which was subsequently synonymized with *Wiedemannia zetterstedti* (Fallén, 1826). However, this is likely a misidentification since the latter species does not occur in this part of Europe and there are substantial taxonomic misidentifications in the *Wiedemannia zetterstedti* “group”. A taxonomic revision of this group of sibling species is ongoing and hopefully the taxonomic status of all species in this complex will be resolved in the near future.

#### Wiedemannia martini

Taxon classificationAnimaliaDipteraEmpididae

Pusch, 1996

467CFA88-5762-5970-979A-36A0DE8259D9

##### Material examined.

• 1♂, 3♀; Zicavo, Ponte di Valpine, near small waterfall in riverbed; 29.vi.2019; HC (2) • 7♂,4♀; Zicavo, Ponte di Valpine, in splash zone of rocks in riverbed; 25.vi.2019; HC (3) • 1♂, 1♀; Zicavo, Ponte di Valpine, on dry rocks and on seepages on rocks in riverbed; 29.vi.2019; HC (4) • 1♂, Serra di Scopamène, Castellu d’Ornucciu, in shady sites along stream in pozzine landscape; 26–30.vi.2019; YPT (11).

##### Remarks.

Recorded and described by Pusch (1996).

### Subfamily Hemerodromiinae

#### Chelifera corsicana

Taxon classificationAnimaliaDipteraEmpididae

Vaillant, 1981

FD0B38B3-736C-53EC-91F0-D3D06A62BDFB

##### Material examined.

• 1♂; Serra di Scopamène et Sorbollano, Campu di Bonza, edge of oak forest; 27.vi.2019; YPT (25).

##### Remarks.

Vaillant (1981) collected and described this species on the basis of a single male. Our specimen represents the second finding of this species.

#### Chelifera precatoria

Taxon classificationAnimaliaDipteraEmpididae

(Fallén, 1815)

BE695C19-CEC0-5980-9646-814143520F1B

##### Material examined.

• 1♂; Zicavo, Ponte di Valpine, on rocks in riverbed; 25–29.vi.2019; YPT (6) • 2♂, 2♀; Serra di Scopamène, Castellu d’Ornucciu, in shady sites along stream in pozzine landscape; 26–30.vi.2019; YPT (11) • 1♂,1♀; Zonza, Samulaghia, on dry rocks near seepage in sapinière forest; 24–28.vi.2019; YPT (15) • 1♂, 1♀; Zonza, Samulaghia, on rocky seepage in sapinière forest (edge of forest); 24–28.vi.2019; YPT (18).

##### Remarks.

Reported previously by Becker et al. (1910).

#### * Chelifera subangusta

Taxon classificationAnimaliaDipteraEmpididae

Collin, 1961

A7CDB5F8-D1D1-5D6E-8207-B6C1B2FFC4E4

##### Material examined.

• 1♂; Zicavo, Ponte di Valpine, at seepage on beech forest slope; 25–29.vi.2019; YPT (8) • 2♂; Zonza, Samulaghia, marshy seepage in dry sapinière forest; 24–28.vi.2019; BPT (20) • 4♂, 16♀; Serra di Scopamène et Sorbollano, Campu di Bonza, on gravelly muddy seepage in deciduous forest; 23–27.vi.2019; BPT (23) • 1♂, 2♀; Serra di Scopamène et Sorbollano, Campu di Bonza, on gravelly muddy seepage in deciduous forest; 23–27.vi.2019; YPT (24).

##### Remarks.

This is the first record of this species from Corsica.

#### * Chelipoda puschae

Taxon classificationAnimaliaDipteraEmpididae

Ivković, Perović & Grootaert sp. nov.

FCA48AB9-714A-5EF5-AE76-08E5FE8174F3

##### Material examined.

•4♂, 1♀; Zicavo, Ponte di Valpine, at seepage on beech forest slope, 25–29.vi.2019; YPT (8) • 1♂, Zicavo, Ponte di Valpine, on rocks on beech forest slope; 25–29.vi.2019; WPT (9) • 2♂, 1♀; Serra di Scopamène, Castellu d’Ornucciu, in higher Alnus forest; 26–30.vi.2019; YPT (10) • 3♂, 2♀; Zonza, Samulaghia, canopied seepage along the road at edge of forest; 24.vi.2019; MSW (13) • 11♂, 3♀; Zonza, Samulaghia, sapinière forest (soil surface); 24–28.vi.2019; YPT (14) • 4♂, 2♀; Zonza, Samulaghia, on dry rocks near seepage in Sapinière forest; 24–28.vi.2019; WPT (15) • 28♂, 15♀; same data; YPT (15) • 18♂, 6♀; Zonza, Samulaghia, sapinière forest; 24–28.vi.2019; YPT (16) • 174♂, 77♀; Zonza, Samulaghia, in dry sapinière forest; 24–28.vi.2019; YPT (17) • 17♂, 14♀; Zonza, Samulaghia, on rocky seepage in sapinière forest (edge of forest); 24–28.vi.2019; YPT (18) • 1♂,1♀; Zonza, Samulaghia, marshy seepage in dry sapinière forest; 24–28.vi.2019; BPT (20) • 1♂, 1♀; same data; WPT (20) • 2♂, 1♀; same data; MSW (20) • 17♂, 23♀; same data; YPT (20) • 7♂, 6♀; Zonza, Samulaghia, on low vegetation in marshy seepage in sapinière forest; 24.vi.2019; MSW (21) • 5♂; Serra di Scopamène et Sorbollano, Campu di Bonza, on banks of river in oak forest; 23–27.vi.2019; YPT (22).

##### Remarks.

See species description above.

#### * Hemerodromia unilineata

Taxon classificationAnimaliaDipteraEmpididae

Zetterstedt, 1842

511AD151-380D-5FE8-8496-086D7FAB09C3

##### Material examined.

• 1♂; Serra di Scopamène et Sorbollano, Campu di Bonza, on banks of river in oak forest; 23–27.vi.2019; YPT (22) • 1♂, Serra di Scopamène et Sorbollano, Campu di Bonza, on gravelly muddy seepage in deciduous forest; 23–27.vi.2019; YPT (24) • 1♂; Serra di Scopamène et Sorbollano, Campu di Bonza, clearing in oak forest; 23.vi.2019; SW (26).

##### Remarks.

This is the first record of this species from Corsica.

### Species richness and assemblage composition

**Figure 6.**
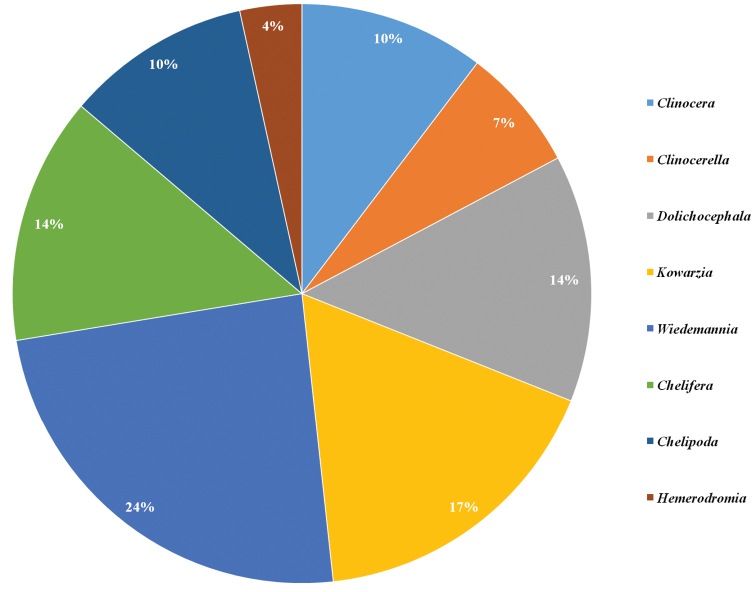
Species richness of aquatic Empididae genera (Diptera: Empididae, Clinocerinae, Hemerodromiinae) on Corsica (total number of species: *Clinocera* – 3 species; *Clinocerella* – 2 species; *Dolichocephala* – 4 species; *Kowarzia* – 5 species; *Wiedemannia* – 7 species; *Chelifera* – 4 species; *Chelipoda* – 3 species; *Hemerodromia* – 1 species).

So far, 29 species of aquatic empidids are recorded from Corsica, France (Table 2). New data on 16 species (12 Clinocerinae and four Hemerodromiinae) and one new hemerodromiine species, *Chelipoda puschae* sp. nov., were retrieved from samples collected at 26 sites during the “La Planète Revisitée Corsica” survey in June 2019 (Fig. 1; Table 1). The subfamily Clinocerinae is represented by 21 species (72%), in five genera: *Clinocera* Meigen (3 species), *Clinocerella* Engel (2 species), *Dolichocephala* Macquart (4 species), *Kowarzia* Mik (5 species), and *Wiedemannia* Zetterstedt (7 species). The subfamily Hemerodromiinae is represented by eight species (28%), in three genera: *Chelifera* Macquart (4 species), *Chelipoda* Macquart (3 species), and *Hemerodromia* Meigen (1 species) (Table 2). The clinocerine genus *Wiedemannia* is the most species-rich (24% of the total number of aquatic empidids on the island), followed by *Kowarzia* (17%) and the genera *Dolichocephala* (14%) and *Chelifera* (14%) (Fig. 6).

**Figure 7.**
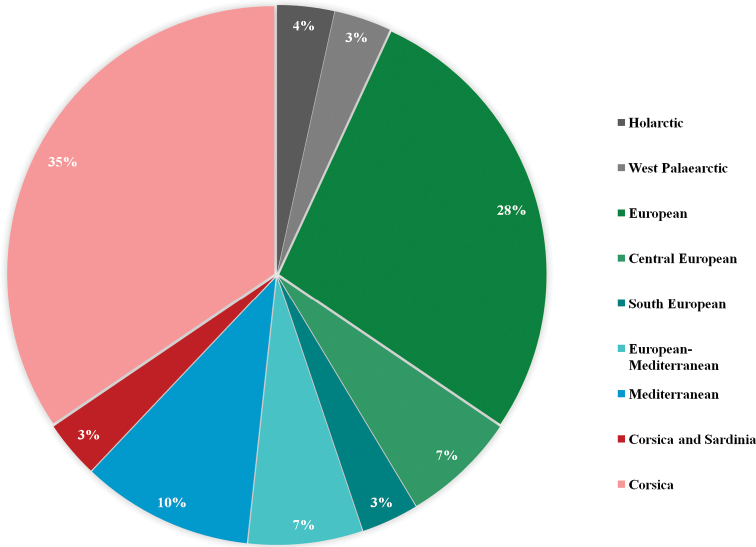
Zoogeographic classification of aquatic empidid species (Diptera, Empididae, Clinocerinae and Hemerodromiinae) currently known from Corsica.

The proportion of endemic species of aquatic empidids in Corsica, i.e. species that have so far only been found in Corsica and that are believed to occur only there, is 35% (10 species discussed here). In addition, *Clinocerella gereckei* (Wagner & Horvat, 1993) also occurs on Sardinia (Italy). Among Corsican species, 28% are widespread European species, 7% are Central European. A single South European species is recorded, *Kowarzia barbatula* (Mik, 1880). *Dolichocephala ocellata* (Costa, 1854) and *Kowarzia bipunctata* (Haliday, 1833) are considered European–Mediterranean (7% of species discussed here), while *Dolichocephala malickyi* Wagner, 1995, *Wiedemannia czernyi* (Bezzi, 1905), and *Chelifera barbarica* Vaillant, 1981 are Mediterranean species (10% of all species). *Clinocera nigra* Meigen, 1804 is a Western Palaearctic species and *Clinocera stagnalis* (Haliday, 1833) a Holarctic species (Fig. 7).

## Discussion

Ten (35%) of the aquatic Empididae recorded from Corsica thus far are considered strictly endemic to the island, and slightly over 40% of the Corsican aquatic empidids are known from other parts of Europe as well (all through Europe, Central Europe, or Southern Europe). The remaining 25% of the species are either widely distributed (Holarctic, Western Palaearctic) or are confined to the Mediterranean area. We compared our list of Corsican species with the existing records of species in Becker et al. (1910), Vaillant (1965, 1982) Wagner (1995), Pusch (1996), Chvála (2012), and Yang et al. (2007). The following six species were not previously recorded from Corsica and represent the first published records: *Dolichocephala malickyi* Wagner, 1995, *D. oblongoguttata* (Dale, 1878), *D. ocellata* (Costa, 1854), *Chelifera subangusta* Collin, 1961, *Hemerodromia unilineata* Zetterstedt, 1842, and *Chelipoda puschae* Ivković, Perović & Grootaert, sp. nov. Moreover, this is the first description of a species of *Chelipoda* from the European–Mediterranean region for more than 180 years.

Of the two subfamilies, the Clinocerinae have a greater species richness in Europe, especially in mountainous areas (Vaillant 1982; Horvat 1995; Ivković et al. 2012, 2013a, 2013b, 2014, 2017, 2020). This agrees with the pattern observed in Corsica and might be explained by the central mountain chain on the island. Likewise, *Wiedemannia* represents the most speciose genus, both in Corsica and on the continent. By contrast, *Chelifera* is usually the second most species-rich genus (Meyer and Wagner 2011; Ivković et al. 2013a, 2013b, 2017, 2020), but in Corsica it is replaced by *Kowarzia*. A higher diversity of *Kowarzia* is usually only present in mountain regions (Ivković et al. 2014).

The aquatic Empididae fauna of Corsica is composed of exclusively Western Palaearctic taxa with the exception of *Clinocera stagnalis* (Haliday, 1833), which is the most widespread Holarctic clinocerine (also known from North America, North Asia, and North Africa) (Sinclair 2008). Most of the Corsican species are restricted to the Central European or Mediterranean regions. However, 10 of the species encountered in Corsica are strictly confined to the island and can therefore be termed endemic. Only five species are shared with the island of Sardinia (Wagner and Horvat 1993; Wagner 1995). We believe that the current species list is far from complete. Indeed, there has not yet been a comprehensive study of Corsica and all of its freshwater habitats. Furthermore, sampling efforts during the “La Planète Revisitée” were restricted to a short period in late spring and only samples from pan traps and sweep net collecting were examined. Some obvious genera such as *Bergenstammia* and *Phaeobalia* are currently absent from the list. Species in these genera are usually found on the continent only above 1,000 m a.s.l., and as most of Corsica is montane, it is our belief that more species, including more endemics, are likely to be found in Corsica. Most endemic freshwater insect species in Corsica are restricted to higher altitudes (500–1,900 m) (Giudicelli 1975). The influence of altitude and isolation on biodiversity processes is more marked in Corsica, with 30 peaks exceeding 2,000 m, than in, for example, Sardinia where the highest mountain is only 1,830 m. This could explain, in part, why Corsica has a seemingly higher overall species richness than Sardinia, including aquatic empidids (only nine species), even though Sardinia is almost triple the size of Corsica (Giudicelli 1975; Chvála 2012). When it comes to aquatic empidids, we have to bear in mind that they may have been collected only sporadically in Sardinia, mostly as a side catch during inventories of other aquatic groups (Wagner 1984, 1995; Wagner and Horvat 1993). Comparisons between the aquatic empidid faunas of Corsica and Sardinia must therefore be made with the utmost caution. However, the greater species richness in Trichoptera, a group with a similar ecological profile to aquatic empidid flies, also suggests a richer fauna in Corsica, with more endemic species in Corsica than in Sardinia (Giudicelli 1975). In addition, most endemics are found at higher altitudes in Corsica than at lower altitudes (Giudicelli 1975). Katmaier and Caccone (2013) have stated that Corsica is faunistically impoverished when compared to continental resources. Our results, on the contrary, suggest otherwise as the number of aquatic empidids is quite high, especially considering the limited sampling efforts. It has been assumed that most of the endemic species that now occur in Corsica have differentiated from ancestors on the Iberian Peninsula (Katmaier and Caccone 2013). In aquatic empidids, however, this might not be the case, as most of the species present are shared with Central and Southern Europe and only a minority is shared with the Iberian Peninsula, but detailed morphological and/or genetic studies could confirm or reject this assumption. It is postulated that during the Messinian Salinity Crisis, the Mediterranean Sea almost completely dried up and a number of freshwater species reached Corsica through an area of braided rivers present all over the Mediterranean and connecting Corsica to the European continent (Katmaier and Caccone 2013).

To conclude, we hope that this paper will assist in the understanding of our present-day knowledge of the aquatic empidids of Corsica and will provide a starting point for further, more detailed and comprehensive studies, as well as additional studies in Sardinia where the aquatic dance fly fauna is poorly known.

## Supplementary Material

XML Treatment for Chelipoda puschae

XML Treatment for Clinocera nigra

XML Treatment for Clinocerella wagneri

XML Treatment for Dolichocephala malickyi

XML Treatment for Dolichocephala oblongoguttata

XML Treatment for Dolichocephala ocellata

XML Treatment for Kowarzia bipunctata

XML Treatment for Kowarzia cataractae

XML Treatment for Kowarzia schnabli

XML Treatment for Kowarzia tibiella

XML Treatment for Wiedemannia corsicana

XML Treatment for Wiedemannia czernyi

XML Treatment for Wiedemannia martini

XML Treatment for Chelifera corsicana

XML Treatment for Chelifera precatoria

XML Treatment for Chelifera subangusta

XML Treatment for Chelipoda puschae

XML Treatment for Hemerodromia unilineata
